# Draft Genome Sequence of the Wood-Degrading Ascomycete *Kretzschmaria deusta* DSM 104547

**DOI:** 10.1128/genomeA.01076-17

**Published:** 2017-10-26

**Authors:** Enrico Büttner, Anna Maria Gebauer, Martin Hofrichter, Christiane Liers, Harald Kellner

**Affiliations:** Department of Bio- and Environmental Sciences, International Institute Zittau, Technische Universität Dresden, Zittau, Germany

## Abstract

We report here the draft genome of *Kretzschmaria* (*Ustulina*)* deusta*, an ascomycetous fungus that colonizes and substantially degrades hardwood and can infest living broad-leaved trees. The genome was assembled into 858 contigs, with a total size of 46.5 Mb, and 11,074 protein-coding genes were predicted.

## GENOME ANNOUNCEMENT

The ascomycete *Kretzschmaria deusta* belongs to the family *Xylariaceae*, which includes fungi causing soft-rot type II, a special type of wood rot (1). These fungi are able to disintegrate cellulose, hemicellulose (mainly xylan), and, to some extent, lignin (2). Unlike basidiomycetes causing white rot, those causing soft rot do not possess ligninolytic class II peroxidases (i.e., manganese-oxidizing peroxidases, lignin peroxidase); instead, they seemingly possess an alternative enzymatic system to degrade substantially woody lignocelluloses. This system has been proposed to consist of numerous and diverse glycoside hydrolases (GHs), several carbohydrate esterases (CEs), and different laccases, which may act jointly in the course of wood degradation and which are of biotechnological interest (2, 3). Whether peroxidative enzymes, such as dye-decolorizing peroxidase (DyP), generic peroxidase (GP), and unspecific peroxygenase (UPO), contribute to lignin modification through wood colonization is still unclear.

The fungus *K. deusta* DSM 104547 (GenBank accession number KY781165) was isolated from a beech stump (*Fagus sylvatica*, Germany, Zittau, Zittauer Gebirge; 50°50′56.1″N, 14°45′39.1″E). The strain was cultivated in 2.5% whey-protein-glucose medium in shake flasks (100 rpm) at 22°C. After harvesting, a freeze-dried biomass sample was used to extract the genomic DNA with the DNeasy plant maxikit (Qiagen, Hilden, Germany). Genomic DNA was enzymatically sheared, and a 200-bp fragment library was prepared using the Ion Xpress Plus fragment library kit (Thermo Fisher, Darmstadt, Germany). After emulsion-based PCR with the Ion PGM template OT2 200 kit, the Ion Torrent PGM was used for sequencing with the Ion PGM 200 version 2 sequencing kit and a 318 V2 chip. Six million reads with a median size of 230 bp were generated.

The (re)assembly of 3.5 million reads, 150 to 380 bp in size, was performed using MIRA version 4.0 (4) integrated in Geneious R10 (5). A second assembly step using the Geneious R10 assembler (with the high-sensitivity setting) was carried out to filter for duplicate contigs. Assembly quality was analyzed using BUSCO (6), and the best assembly showed 94.2% completeness of full-length conserved genes (predictor, *Aspergillus oryzae*). Genome statistics were evaluated using QUAST (7). The draft genome sequence of *K. deusta* was assembled into 885 contigs, with a total size of 46.5 Mb, an *N*_50_ value of 89,981, and an average G+C content of 47.1%. The genome was annotated using AUGUSTUS (species parameter, *A. oryzae*) (8), and a total of 11,074 protein-coding sequences were predicted. Specific enzymes of interest were annotated and filtered using Blast2GO (BioBam, Valencia, Spain) and dbCAN (9).

Altogether, 696 carbohydrate-related enzymes and modules were identified: 297 glycoside hydrolases (including 4 GH78s), 104 carbohydrate esterases, 15 polysaccharide lyases, 82 glycosyltransferases, 131 enzymes with auxiliary activities, and 67 carbohydrate-binding modules (CBMs), among them 13 from CBM family 1, which binds to cellulose. Several oxidoreductases with putative activities toward aromatic compounds—namely, three UPOs (members of the heme-thiolate peroxidase superfamily) (10), two DyPs (subfamilies A and D) (11), and two class II peroxidases (i.e., GPs) (12)—were identified as members of the plant-fungal-bacterial peroxidase superfamily (11) (GenBank accession numbers KY782128 to KY782142).

### Accession number(s).

This whole-genome shotgun project has been deposited at DDBJ/ENA/GenBank under the accession number MLHU00000000. The version described in this paper is the third version, MLHU03000000.
